# A Variant Form of the Human Deleted in Malignant Brain Tumor 1 (DMBT1) Gene Shows Increased Expression in Inflammatory Bowel Diseases and Interacts with Dimeric Trefoil Factor 3 (TFF3)

**DOI:** 10.1371/journal.pone.0064441

**Published:** 2013-05-15

**Authors:** Jens Madsen, Grith Lykke Sorensen, Ole Nielsen, Ida Tornøe, Lars Thim, Claus Fenger, Jan Mollenhauer, Uffe Holmskov

**Affiliations:** 1 Sir Henry Wellcome Laboratories, Department of Child Health, Clinical and Experimental Sciences, Faculty of Medicine, Southampton General Hospital, University of Southampton, Southampton, United Kingdom; 2 Institute for Life Sciences, University of Southampton, Southampton, United Kingdom; 3 Institute of Molecular Medicine, University of Southern Denmark, Odense, Denmark; 4 Department of Pathology, Odense University Hospital, Odense, Denmark; 5 Department of Protein Engineering, Novo Nordisk A/S, Måløv, Denmark; 6 Lundbeckfonden Center of Excellence NanoCAN, University of Southern Denmark, Odense, Denmark; St. Jude Children's Research Hospital, United States of America

## Abstract

The protein deleted in malignant brain tumors (DMBT1) and the trefoil factor (TFF) proteins have all been proposed to have roles in epithelial cell growth and cell differentiation and shown to be up regulated in inflammatory bowel diseases. A panel of monoclonal antibodies was raised against human DMBT1^gp340^. Analysis of lung washings and colon tissue extracts by Western blotting in the unreduced state, two antibodies (Hyb213-1 and Hyb213-6) reacted with a double band of 290 kDa in lung lavage. Hyb213-6, in addition, reacted against a double band of 270 kDa in colon extract while Hyb213-1 showed no reaction. Hyb213-6 showed strong cytoplasmic staining in epithelial cells of both the small and large intestine whereas no staining was seen with Hyb213-1. The number of DMBT1^gp340^ positive epithelial cells, stained with Hyb213-6, was significantly up regulated in inflammatory colon tissue sections from patients with ulcerative colitis (p<0.0001) and Crohn’s disease (p = 0.006) compared to normal colon tissue. Immunohistochemical analysis of trefoil factor TFF1, 2 and 3 showed that TFF1 and 3 localized to goblet cells in both normal colon tissue and in tissue from patients with ulcerative colitis or Crohn’s disease. No staining for TFF2 was seen in goblet cells in normal colon tissue whereas the majority of tissue sections in ulcerative colitis and Crohn’s disease showed sparse and scattered TFF2 positive goblet cells. DMBT1 and TFF proteins did therefore not co-localize in the same cells but localized in adjacent cells in the colon. The interaction between DMBT1^gp340^ and trefoil TFFs proteins was investigated using an ELISA assay. DMBT1^gp340^ bound to solid-phase bound recombinant dimeric TFF3 in a calcium dependent manner (p<0.0001) but did not bind to recombinant forms of monomeric TFF3, TFF2 or glycosylated TFF2. This implies a role for DMBT1 and TFF3 together in inflammatory bowel disease.

## Introduction

The gene *Deleted in Malignant Brain Tumors* 1 (DMBT1) was identified more than a decade ago as a region of chromosome 10q25.3-q26.1 based on homologous deletions and lack of expression in medulloblastoma, glioblastoma multiforme, lung and gastrointestinal cancers and has been suggested as a potential tumor-suppressor gene [1]–[5]. The *DMBT1* gene encodes extracellular secreted molecules with the longest variant featuring 13 scavenger receptor cysteine-rich (SRCR) domains separated by SRCR interspersed domains (SIDs) [6].

The domain organization of DMBT1 indicates that CRP-ductin [7], ebnerin [8] and hensin [9] are ortholog proteins in mouse, rat and rabbit, respectively and the genomic structure and organization further supported this view [10], [11].

DMBT1 and CRP ductin are localized in epithelial cells on mucosal surfaces and in skin that suggests a role for DMBT1 in epithelial development [6], [7], [12], [13]. This was supported by other studies showing that hensin and ebnerin have a possible role in the activation and differentiation of epithelial cells [14], [15].

Besides the roles involved in cell differentiation and growth, a role for DMBT1 in innate immunity has also been suggested. The glycoprotein 340, gp-340, was purified from human bronchoalveolar lavage with a molecular weight of 340 kDa under reducing SDS-PAGE conditions, where it was observed as a double band (named as band A and B) [16]. Gp-340 was shown to interact specifically with the collectin proteins surfactant protein A and D (SP-A and -D), both are molecules involved in the mucosal immune defense system [16], [17]. Sequence data showed that gp-340 is encoded by the *DMBT1* gene, corresponding to the longest variant and hence the protein was named DMBT^gp340^ [6]. DMBT1^gp340^ was shown to be identical to salivary agglutinin [18], a glycoprotein isolated from parotic saliva implicated in the protection against dental caries through its binding and agglutination of *Streptococcus mutans* [19] and hence named DMBT1^SAG^. DMBT1 proteins bind to other microorganisms like Helicobacter pylori, several strains of streptococcus and viruses including *Human Immunodeficiency* Virus Type 1 and influenza A virus [18], [20], [21]. The bacterial binding site has been confined to the SRCR domain [22], [23] The broad pathogen-specificity of DMBT1 is, at least in part, based on it acting as pattern recognition receptor for repeated phosphate and sulfate groups [24]. It seems therefore likely that DMBT1 proteins also contribute to innate defense against bacteria and viruses in the gastrointestinal and respiratory tracts through direct interactions with microorganisms and/or through interactions with SP-A or SP-D.

The two most common inflammatory bowel diseases (IBD) are ulcerative colitis (UC) and Crohn’s disease (CD). The etiology for both diseases is unknown but is considered to be multifactorial; both involving the environment, the commensal gut flora and the genetic makeup of an individual (recently reviewed in [25]). The small intestine is most commonly affected by CD but any part of the gastrointestinal tract can be involved whereas only the colon and rectum are normally affected by UC. DMBT1 has been found to be up regulated in an *ex-vivo* model using primary intestinal epithelial cells and proinflammatory stimuli such as tumor necrosis factor alpha and LPS from *Salmonella enterica* [26]. DMBT1 is also up regulated *in vivo* in the small intestine and colon, shown in intestinal biopsy samples from patients diagnosed with inflammatory bowel disease such as UC and CD [26], [27]. This was measured on the mRNA level by quantitative real-time PCR and shown on the protein level as well as by immunohistochemistry using a rabbit polyclonal antibody raised against full-length recombinant DMBT1 [26], [27]. That DMBT1 has a direct involvement in inflammatory bowel diseases *in vivo* was shown in a mouse model where mice made deficient for DMBT1 were found to have an enhanced susceptibility to dextran sulfate sodium (DSS) induced colitis [27].

Porcine DMBT1 isolated from the stomach has been identified as a possible binding molecule for the trefoil factor 2 (TFF2) [28]. Trefoil factors are a family of three members (TFF1, 2 and 3). They are small compact proteins with trefoil motifs and an ability to cross-link mucous and glycoproteins [29]. Cloning of human TFF2 showed that TFF2 contained two TFF motifs and recombinant expression in a yeast system showed that TFF2 is expressed in a dimeric state but with or without an asparagine N-linked glycosylation [30], [31]. Cloning of human TFF3 showed that it contains one TFF motif but recombinant expression in a yeast system showed that the protein is can be purified as either a monomeric or dimeric molecule where the latter is composed of two monomers linked with a disulphide bridge [32], [33]. TFF3 is expressed in mucus-secreting cells on mucosal surfaces throughout the human body, e.g. goblet cells in the colon, with a tissue profile similar to SP-D and DMBT1^gp340^, whereas TFF1 and TFF2 show a more restricted tissue localization [34]. Mice deficient for each of the TFFs, made by gene-targeting disruption, all showed a phenotype of decreased ability to regenerate mucosal healing after injury by numerous inflammatory agents [35]–[37]. This implied that TFF proteins play a protective role, with a function in regeneration of mucosal epithelia. This was supported by the finding that with a transgenic mouse mode overexpressing TFF2, the mice showed an increased resistance to intestinal damage [38]. All TFF proteins have been found to be up regulated, on the RNA level, in inflammatory bowel diseases like CD or UC (reviewed in [39]).

Here we present data showing that the number of DMBT1^gp340^ positive epithelial cells is up regulated in the inflammatory bowel diseases CD and UC. Furthermore, we found that DMBT1^gp340^ binds specifically to recombinant human dimeric TFF3 but not to recombinant proteins of human glycosylated or non-glycosylated TFF2 or monomeric TFF3.

## Materials and Methods

### Ethics Statement

Human Tissue used for analysis by SDS-PAGE/Western blotting and immunohistochemistry.

Human tissues samples were from the tissue bank at the Department of Pathology, Odense University Hospital, Denmark. The Regional Scientific Ethical Committee for Southern Denmark approved the use of human tissue samples for research purposes (VF20050070) and samples were obtained from patients with written informed consent. Normal human colon tissue was obtained from patients undergoing resection for carcinoma of the colon and samples were taken from regions several centimeters away from the tumor. Colon tissue from patients with inflammatory bowel disease was obtained from patients undergoing resection for diagnostic or therapeutic purposes. Bronchoalveolar lavage (BAL) was obtained from patients with written informed consent undergoing lung washings for therapeutic purposes including pulmonary alveolar proteinosis. The procedure was approved by the London National Health Service Research Ethics Committee.

### Buffers and Reagents

Alkaline phosphatase-conjugated goat anti-mouse IgG (whole molecule) antibody (Sigma-Aldrich, St. Louis, MO, USA); coating buffer: 60 mM Na_2_CO_3_, 35 mM NaHCO_3_, 0.02% (w/v) NaN_3_, pH 9.6; diethanolamine buffer: 10% (v/v) diethanolamine, 0.5 mM MgCl_2_, 0.02% (w/v) NaN_3_, pH 8.9; *p*-nitrophenylphosphate, disodium salt (PNPP) (Boehringer Mannheim, Mannheim, Germany); phosphate buffered saline (PBS): 123 mM NaCl, 10 mM sodium phosphate, 3 mM potassium phosphate, 0.02% (w/v) NaN_3_, pH 7.4; PBS/BSA: PBS containing 0.1% (w/v) BSA; sample buffer: 1.5% (w/v) sodium dodecylsulfate (SDS), 5% (v/v) glycerol, 0.2% (w/v) bromphenol blue, 0.1 M Tris, pH 8.0; SSC (20x): 3 M NaCl, 3.65 M sodium citrate, pH 7.2; substrate buffer: 100 mM NaCl, 100 mM Tris, 5 mM MgCl_2_; pH 9.5. Tris-buffered saline (TBS): 140 mM NaCl, 10 mM Tris-HCl, 0.02% (w/v) NaN_3_, pH 7.4; TBS/Tw: TBS containing 0.05% (v/v) Tween 20 (polyoxyethylene sorbitan monolaurate, Merck Eurolab, Darmstadt, Germany); WB buffer: TBS/Tw with a final conc of 0.5 M NaCl. All procedures took place at room temperature unless otherwise stated.

### SDS-PAGE and Western Blotting of BAL and Colon Homogenates

The PhastSystem (Amersham Pharmacia Biotech, Uppsala, Sweden) was used for SDS-PAGE and Western blotting. Tissue and cells were homogenized in TBS with 10 mM EDTA, 10 mM Tris, 5 mM iodoacetamide, 5 mM trans-4-(aminomethyl) cyclohexanecarboxylic acid (Sigma-Aldrich), 1 µg/mL Aprotinin (Boehringer Mannheim) and 1% (v/v) Triton X-100. The homogenates were centrifuged at 10,000×g for 30 min at 4°C. The supernatants were separated on 4–15% (w/v) polyacrylamide gradient gels with the discontinuous buffer system. Samples were reduced by heating at 100°C for 3 min in 40 mM dithiothreitol, 1.5% (w/v) SDS, 5% (v/v) glycerol, 0.1 M Tris, pH 8.0, and carboxamidated by the addition of iodoacetamide to a concentration of 90 mM. Unreduced samples were heated in sample buffer with 90 mM iodoacetamide. After the electrophoresis proteins were blotted onto polyvinylidene difluoride membranes (Immobilon P, Millipore, Bedford, MA, USA). The membranes were blocked for 30 min in 0.1% Tween 20 in H_2_O followed by overnight incubation at 4°C with the primary antibody (Hyb213-1 or Hyb213-6) at a concentration of 50 ng/mL in WB buffer. The membranes were washed three times in WB buffer and then incubated with a secondary alkaline-phosphatase-coupled rabbit anti-mouse IgG antibody (diluted1∶1000, Dako) in WB buffer. The membranes were washed three times in WB buffer, followed by one time in TBS/Tw and developed in substrate buffer with nitro blue tetrazolium and potassium 5 bromo-4-chloro-3-indolylphosphate.

### Immunohistochemistry

Four µm sections were cut from neutral-buffered formaldehyde-fixed paraffin embedded tissue blocks. Sections were mounted on ChemMate Capillary Gap Slides (Dako, Glostrup, Denmark), dried at 60°C, deparaffinized and hydrated.

DMBT1^gp340^: Antigen retrieval was performed using microwave heating in Target Retrieval Solution (Dako). Three Tissue-Tek containers (Miles Inc., Elkhart, IN, USA), each with 24 slides in 250 mL buffer, were placed on the edge of a turntable inside the microwave oven. Slides were heated 11 min at full power (900W), then for 15 min at 400W. After heating, slides remained in the buffer for 15 min. Antigen retrieval was followed by blocking of endogenous biotin, using Dako’s Biotin-Blocking System (Dako). Incubation with Hyb213-6 (625 ng/mL) was done for 25 min.

TFF proteins: Tissue sections for TFF1, TFF2 and TFF3 were stained according to procedures describes previously [34]. Shortly, antigen retrieval was performed for TFF1 and TFF3 as described for DMBT1^gp340^, whereas antigen retrieval for TFF2 was performed using 0.05% protease type XIV (pronase E, cat. #P5147; Sigma-Aldrich) in TBS, pH 7.0, for 15 min at 37^o^C. Polyclonal rabbit antisera (stock conc 1 mg/mL) against TFF1 (2239A), TFF2 (2240A), and TFF3 (2241A) were used at dilutions 1∶2000, 1∶1000 and 1∶10,000, respectively.

Immunostaining of all sections was automated using the ChemMate horseradish peroxidase/3,3′-diaminobenzidine tetrahydrochloride detection kit (HRP/DAB, K5001; Dako) on the TechMate 1000 instrument (Dako). Immunostaining was followed by brief nuclear counter staining in Mayer’s hematoxylin. Finally, cover slips were mounted with AquaTex (Merck Eurolab).

Controls were performed by replacing the primary monoclonal antibody with an irrelevant monoclonal antibody of the same subclass as the DMBT1^gp340^ antibodies or no primary antibody for the polyclonal TFF antibodies.

The DMBT1^gp340^ staining of normal healthy tissue and inflammatory colon diseased tissue (CD and UC) were quantified with a score of 1 for a staining pattern seen in normal healthy tissue (<25% of the epithelial cells), 2 for staining of 50% of epithelial cells and 3 for staining of 100% of epithelial cells. Scoring was performed independently and in a blinded manner by two researchers (JMa and ON) and one pathologist (CF). Means and standard derivation (SD) was calculated for each group (normal tissue, CD and UC).

### Purified Native DMBT1^gp340^ and Recombinant TFF Proteins

Native DMBT1^gp340^ was purified from bronchoalveolar lavages from patients with pulmonary alveolar proteinosis as described previously [16]. All TFF proteins used, glycosylated and non-glycosylated hTTF2 and monomeric and dimeric hFFT3, were recombinant proteins expressed in a yeast system and purified as described previously [31], [33]. Reduced samples were analyzed by SDS-PAGE as described above, except samples were analyzed on 4–12% (w/v) Novex Tris-Glycine polyacrylamide gradient gels with the discontinuous buffer system according to manufactures instructions (Invitrogen). SDS-PAGE gels with reduced DMBT1^gp340^ was silver stained as described before [40] and reduced TFF proteins were stained using SimplyBlue SafeStain according to manufactures instructions (Invitrogen).

### Binding of Human Recombinant Trefoil Factors to gp-340

All trefoil peptides used, glycosylated and non-glycosylated hTTF2 and monomeric and dimeric hFFT3, were recombinant proteins expressed in a yeast system and purified as described previously [31], [33]. Microtiter plates (Maxisorp, Nunc, Kamstrup, Denmark) were coated with serial two-fold dilutions from 2 µg/mL of recombinant forms of TFFs in coating buffer (0.1 M sodium carbonate, pH 9.6) overnight at 4^o^C in a humidified chamber. This incubation and all the following steps were carried out in a volume of 100 µL/well and at room temperature unless specified. Washes and incubations were carried out four times with TBS with 0.05% Tween20 and 5 mM CaCl_2_ (TBS/Tw). The plates were washed with and blocked for non-specific binding in 200 µL of TBS/Tw for 2 h. After washing, the plates were incubated with two-fold serial dilutions starting from 600 ng/mL of DMBT1^gp340^ for 2 h. After washing, plates were incubated with a rabbit polyclonal anti-DMBT1^gp340^ antibody (K421) 10 µg/mL in TBS/Tw for 2 h. A negative control for the calcium dependent binding was performed with DMBT1^gp340^ in TBS/Tw and 10 mM EDTA. The plates were washed and incubated for 2 h with alkaline phosphatase-coupled goat anti-rabbit IgG (Sigma-Aldrich, cat no A8025) diluted 1∶2000 for one hour. After a final wash, the bound enzyme was estimated by adding 1 µg/mL of 4-Nitrophenyl phosphate (PNPP) in substrate buffer (10% (v/v) diethanolamine, 0.5 mM MgCl_2_, pH 9.8) in the dark until sufficient color development had occurred. The absorbance of the 96 wells was read at 405 nm by means of a multichannel spectrophotometer. Each sample was done in duplicate and performed independently three times. Means and standard derivation (SD) was calculated for each sample.

### Statistics

The nonparametric statistical Kruskal-Wallis test was used to compare means between groups. This was analyzed using the software GraphPad Prism version 6. A p value less than 0.05 was considered statistically significant.

## Results

We have previously shown by RT-PCR that the main sites of synthesis of DMBT1^gp340^ are the trachea, salivary gland, small intestine, lung and stomach [6]. Immunohistochemical analysis using Hyb213-1 as primary antibody revealed staining of alveolar macrophages in the lung but no staining in the small intestine that could justify the strong signal seen by RT-PCR analysis. In contrast, a rabbit polyclonal antibody raised against DMBT1^gp340^ (K421) reacted strongly with epithelial cells of the small intestine and with alveolar macrophages in the lung and weakly with alveolar type II cells [6].These results prompted us to make a new round of monoclonal antibodies directed against DMBT1^gp340^.

### Specificity of the Monoclonal Antibodies Hyb213-1 and Hyb213-6 in Western Blotting of Lung Washings and Colon Tissue Extracts

**Figure 1:**
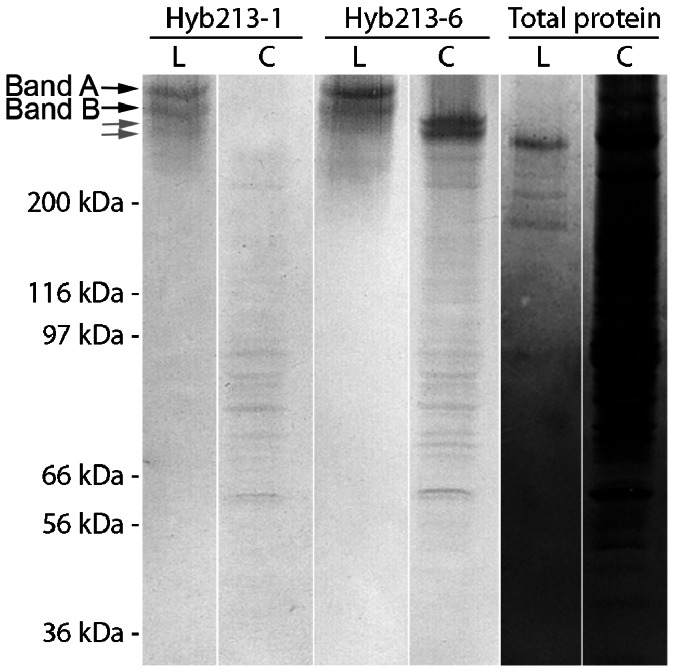
SDS-PAGE and Western blot of lung washing (L) and colon tissue (C) in the non-reduced state. The blots were incubated with the two monoclonal antibodies Hyb213-1, Hyb213-6 or stained for total protein content as described in the Materials and Methods section. Black arrows: Double band (band A and B) of DMBT1^gp340^ from bronchoalveolar lavage. Grey arrows: Double band of DMBT1 from colon tissue.

The specificity of the monoclonal antibodies against DMBT1^gp340^ was analyzed by Western blots of crude lung lavage and colon tissue extract separated on SDS-PAGE in the unreduced state. Hyb213-1 reacted with both bands of 340 kDa equally well in the reduced state (not shown) and with the double band of 290 kDa seen in the unreduced state in lung washings **(** **Fig. 1** **)**. No reaction was seen when analyzing colon extract **(** **Fig. 1** **)**. Hyb213-6 directed against DMBT1^gp340^ reacted with the same double band of 290 kDa in the unreduced state in lung washings and in the colon extract. Hyb213-6 reacted with a double band of approximately 270 kDa in the non-reduced state **(** **Fig. 1** **)**. Hyb213-6 does not react with DMBT1^gp340^ in the reduced state in any tissue extracts analyzed (not shown). When analyzing detergent solubilized alveolar macrophages from BAL and BAL in parallel from the same person in the unreduced state, a double band of approximately 290 kDa was observed with both monoclonal antibodies (not shown). In saliva, both antibodies recognized the same double band in the unreduced state as seen in the lung (not shown).

### Tissue Localization of gp-340 Using Hyb213-1 and 213-6 for Immunohistochemistry

**Figure 2:**
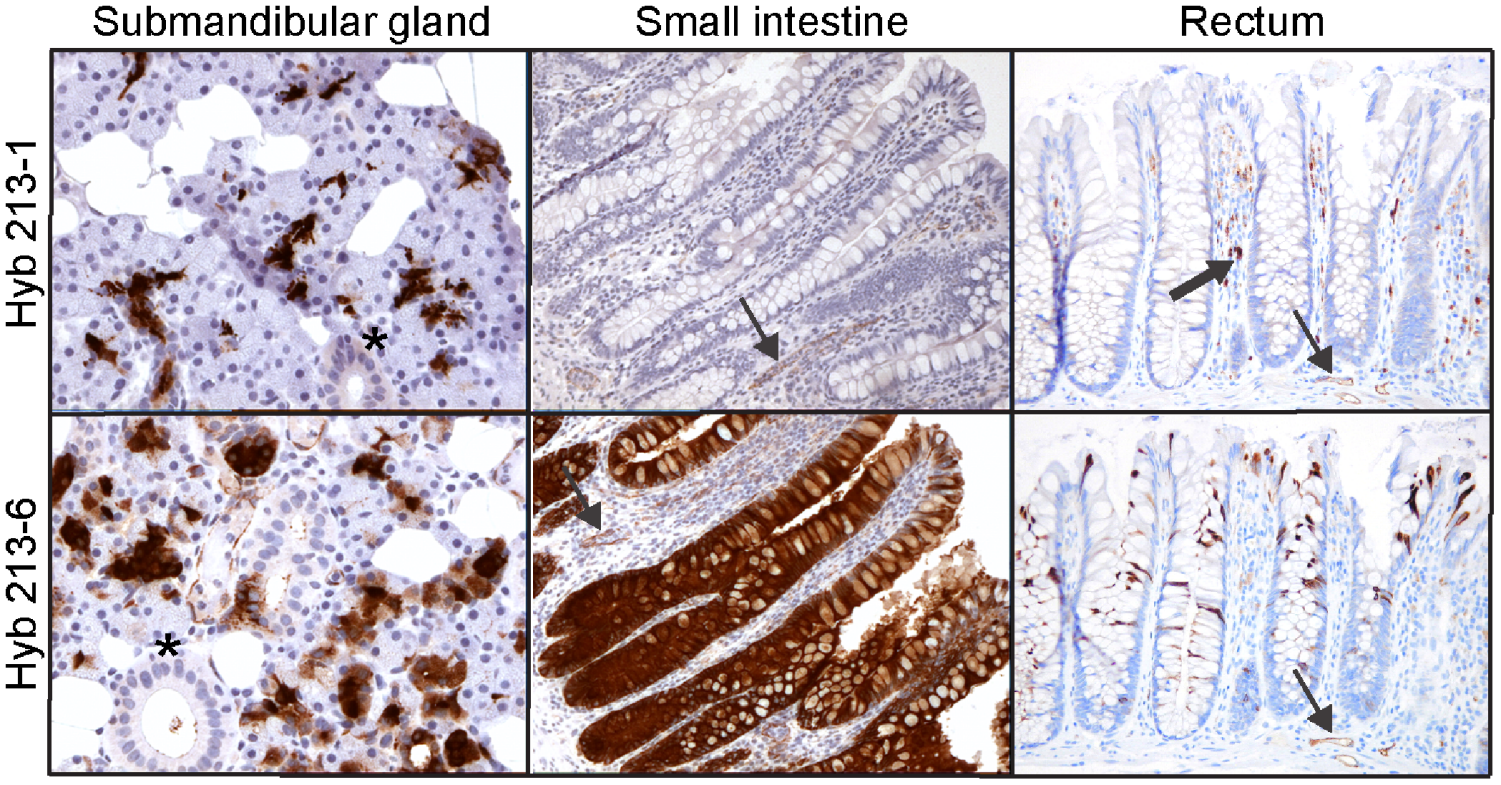
Immunohistochemical localization of DMBT1^gp340^ in human submandibular glands, small intestine and rectum. The tissues were stained using monoclonal antibodies Hyb213-1 or Hyb213-6 directed against DMBT1^gp340^ and an indirect immunperoxidase technique and counterstained with Mayer’s hematoxylin as described in the Materials and Methods section. A positive signal is shown by brown color. Asterisks show striated ducts. Thin black arrow shows stained endothelial cells. Thick black arrow shows a stained macrophage in the lamina propia Original magnification: 100×.

Antigen retrieval was performed on normal human tissues using microwave heating in Target Retrieval Solution. This technique was more sensitive compared to the previous methodology where citrate buffer was used as the antigen retrieval buffer [6]. Strong immunostaining was seen in the serous acini and demilune cells of the submandibular gland when both monoclonal antibodies Hyb213-1 and Hyb213-6 were used for immunohistochemical analysis (**Fig. 2**). Strong staining was also observed with Hyb213-6 in the tall columnar epithelial cells covering the villi and increasing in intensity towards the bottom of crypts, including the crypts of Lieberkühn in the small intestine, while no staining of epithelial cells was observed when using Hyb213-1 **(** **Fig. 2** **)**. Macrophages in lamina propia of the intestine were clearly stained by Hyb213-1 while Hyb213-6 showed less intense staining. Both antibodies also stained endothelia cells in all tissue sections analyzed. Hyb213-6 reacted equally well with the adsorptive crypt and luminal epithelial cells in the colon and rectum while Hyb213-1 did not recognize these epithelial cells **(** **Fig. 2** **)**.

### DMBT1^gp340^ is Up Regulated in Colon Epithelial Cells from Patients with Inflammatory Bowel Disease

**Figure 3:**
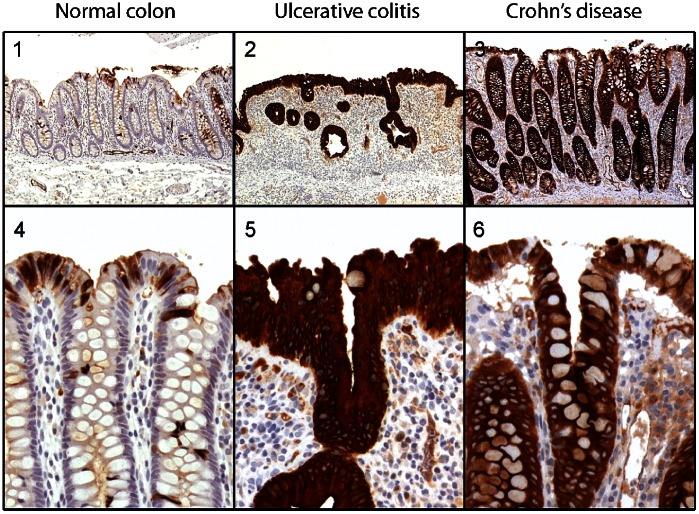
Immunohistochemical localization of DMBT1^gp340^ in normal human colon and inflammatory bowel diseases ulcerative colitis and Crohn’s disease. The tissue sections were stained using the monoclonal antibody Hyb213-6 directed against DMBT1^gp340^ and an indirect immunperoxidase technique and counterstained with Mayer’s hematoxylin as described in the Materials and Methods section. A positive signal is shown by brown color. Original magnification: 1–3∶50x; 4–6∶200x.

Immunohistochemical analysis was performed on normal colon tissue (n = 13) and inflamed colon tissue from 18 patients diagnosed with UC and 10 patients diagnosed with CD using Hyb213-6. The number of DMBT1^gp340^ positive epithelial cells was evaluated in segments of inflamed tissue in all patients and compared with normal colon tissue. Normal colon tissue (n = 13) showed clear but scattered staining of the absorptive epithelial cells along the length of crypts in the colon in ten out of 13 samples **(** **Fig. 3** **)**. All patients diagnosed with UC (n = 18) or CD (n = 10), except two patients in the UC group and one patient in the CD group, showed uniformly strong cytoplasmic staining of epithelial cells along the entire length of crypts in the colon (**Fig. 3B**). The three patients that did not show strong cytoplasmic staining showed moderately increased staining along the length of crypts in the colon (data not shown).

The number of DMBT1^gp340^ positive epithelial cells was quantified using a simple scoring matrix: A staining pattern seen in normal healthy colon tissue (<25% of the epithelial cells) gave a score of 1 whereas staining of approximately 50% of the cells gave a score of 2 and staining of 100% of the epithelial cells gave as score of 3. Using this scoring matrix, normal healthy colon tissue had a mean (±standard derivation) of 1.462 (±0.877) whereas CD and UC had means of 2.900 (±0.316) and 2.889 (±0.323), respectively (**Table 1**). Statistical analysis showed that the number of DMBT1^gp340^ positive cells seen in CD and UC tissue sections were statistically significantly increased when compared to normal healthy tissue (normal vs CD: p = 0.0006; normal vs UC: p<0.0001, **Table 1**). No statistically significant difference was found between the number of DMBT1^gp340^ positive cells in CD and UC (p>0.9999, **Table 1**).

**Table 1:** Quantification of the number of DMBT1^gp340^ positively stained epithelial cells in normal human colon tissue and inflammatory bowel diseases.

Tissue	Numberof samples	Scoring Matrix			Mean	SD	Statistics
		1	2	3			
Normal	13	10	0	3	1.462	0.877	Normal vs CD: p = 0.0006*
CD	10	0	1	9	2.900	0.316	Normal vs UC: p<0.0001*
UC	18	0	2	16	2.889	0.323	CD vs UC: p>0.9999

Normal colon tissue and inflamed ulcerative colitis (UC) or Crohn’s disease (CD) colon tissue was stained for DMBT1^gp340^ using the monoclonal antibody Hyb213-6. The number of DMBT1^gp340^ positive epithelial cells was quantified using a score of 1 for a staining pattern seen in normal healthy tissue (<25% of the epithelial cells), 2 for staining of 50% of epithelial cells and 3 for staining of 100% of epithelial cells. Mean and standard derivation (SD) was calculated for each group: Normal, Crohn’s disease (CD) and ulcerative colitis (UC). Statistical analysis was performed using the nonparametric statistical Kruskal-Wallis test. A p value less than 0.05 was considered statistical significant and marked with an asterisk.

### TFF Staining in Colon Epithelial Cells from Patients with Inflammatory Bowel Disease

**Figure 4:**
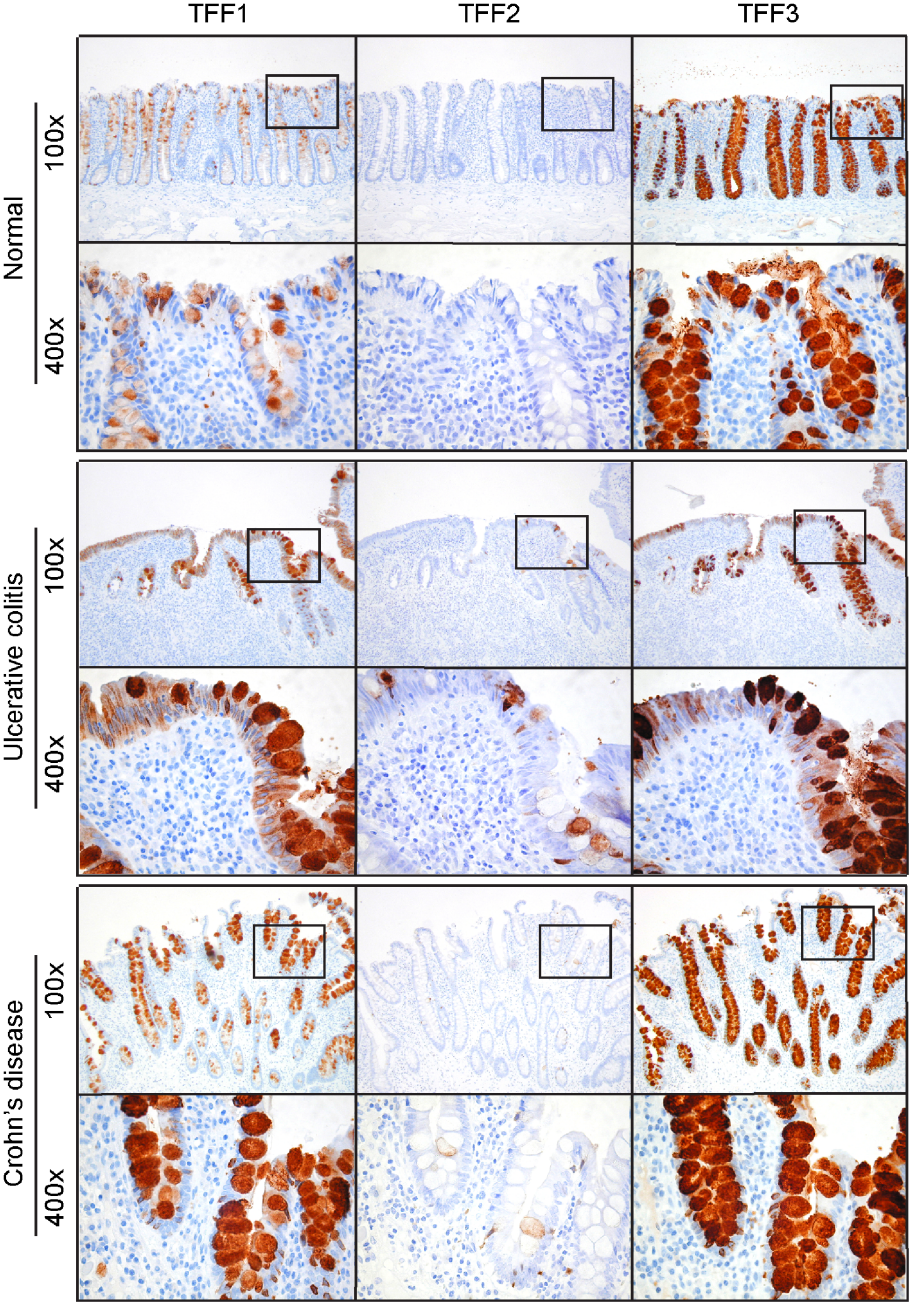
Immunohistochemical localization of TFF1, 2 and 3 proteins in normal human colon and the inflammatory bowel diseases ulcerative colitis and Crohn’s disease. Tissue sections were stained using polyclonal antibodies raised against TFF1, TFF2 and TFF3 and an indirect immunperoxidase technique and counterstained with Mayer’s hematoxylin as described in the Materials and Methods section. A positive signal is shown by brown color. Original magnification: 100× and 400×. Black squares in 100× pictures show approximately location of 400× pictures.

As TFF proteins have been suggested as a putative receptor for porcine stomach DMBT1, we performed immunohistochemical analysis of the same tissue sections as for DMBT1^gp340^ with the following groups: Normal (n = 13), CD (n = 10) and UC (n = 18). These were stained using polyclonal antibodies against TFF1, TFF2 and TFF3 as described previously [34]. TFF1-positive goblet cells were found in 13 out of 13 normal tissue sections and for all UC sections (n = 18) and DC sections (n = 10). The TFF1 staining was seen as a gradient of staining intensity with no staining at the bottom of the crypts, increasing towards the lumen for all normal tissue sections and for all sections in both the CD and UC groups (**Fig. 4**). No TFF2 positive goblet cells were seen in any normal colon tissue (n = 13) whereas most of the IBD tissue sections contained scattered individual TFF2 positive goblet cells (UC 12 out of 18; CD 8 out of 10) (**Fig. 4**). All goblet cells from the base of the crypts to the epithelial surface at the top and onto the luminal surface were stained positive for TFF3 in normal human colon tissue (n = 13), as well as in both groups for CD (n = 10) and UC (n = 18) (**Fig. 4**).

As trefoil proteins have been reported to be up regulated in inflammatory bowel diseases on the mRNA level compared to normal tissue we wanted to see if this could be seen at the protein level using the immunohistochemical staining methodology. A dilution series of the TFF3 antibody was performed to find a dilution with a weak, but discrete, staining in normal tissue that would allow an easy visualization of up regulated TFF3 staining in inflamed CD and UC colon tissues. The TFF3 antibody was diluted from 1∶10,000 to 1∶3,200,000 times (**Supplementary fig. S1**). Two dilutions of 1∶400,000 and 1∶600,000 were chosen and these were applied to all normal colon tissue sections (n = 13) and the two groups of CD (n = 10) and UC (n = 18) inflamed colon tissue sections. However, when using this methodology it was not possible to see any difference in the staining intensity between normal tissue sections and inflamed CD or UC tissue sections as the staining intensity varied within single tissue sections (**Supplementary fig. S1**).

### SDS-PAGE of Purified Native DMBT1^gp340^ and Recombinant TFF Proteins

**Figure 5:**
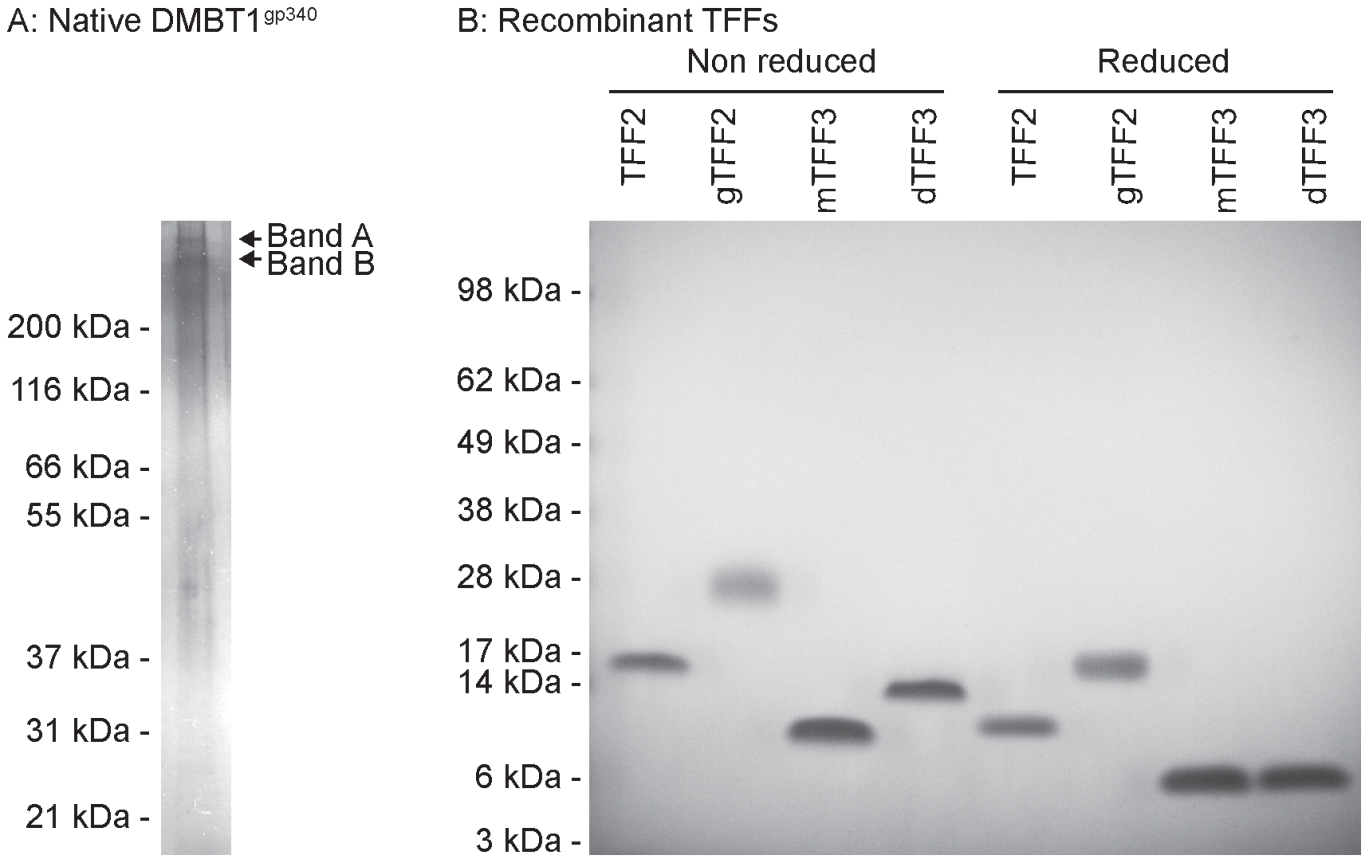
SDS-PAGE of purified native DMBT1^gp340^ from BAL and recombinant TFF proteins. A) Purified native DMBT1^gp340^ in the reduced state visualized using silver staining. B) Purified non reduced and reduced recombinant TFF proteins staining using SafeStain staining. gTFF2: Glycosylated TFF2, mTFF3: Monomeric TFF3 and dTFF3: Dimeric TFF3.

Reduced SDS-PAGE of native DMBT1^gp340^ purified from BAL from patients with pulmonary alveolar proteinosis visualized by silver staining showed a double band (band A and B) migrating with a molecular weight around 300–350 kDa (**Fig. 5A**). Non reduced and reduced SDS-PAGE of purified recombinant TFF proteins and visualized by a coomassie-based staining showed a single distinctive band for all TFF proteins (**Fig. 5B**). Non-glycosylated TFF2 migrated with a molecular weight of approximately 10 kDa and glycosylated TFF2 with a weight of 15 kDa. Dimeric TFF3 migrated with a higher molecular weight than monomeric TFF3 in the non-reduced state but both monomeric and dimeric TFF3 migrated with a molecular weight of approximately 6–7 kDa in the reduced state (**Fig. 5B**).

### Binding of DMBT1^gp340^ to Solid-phase Human Trefoil Factors

**Figure 6:**
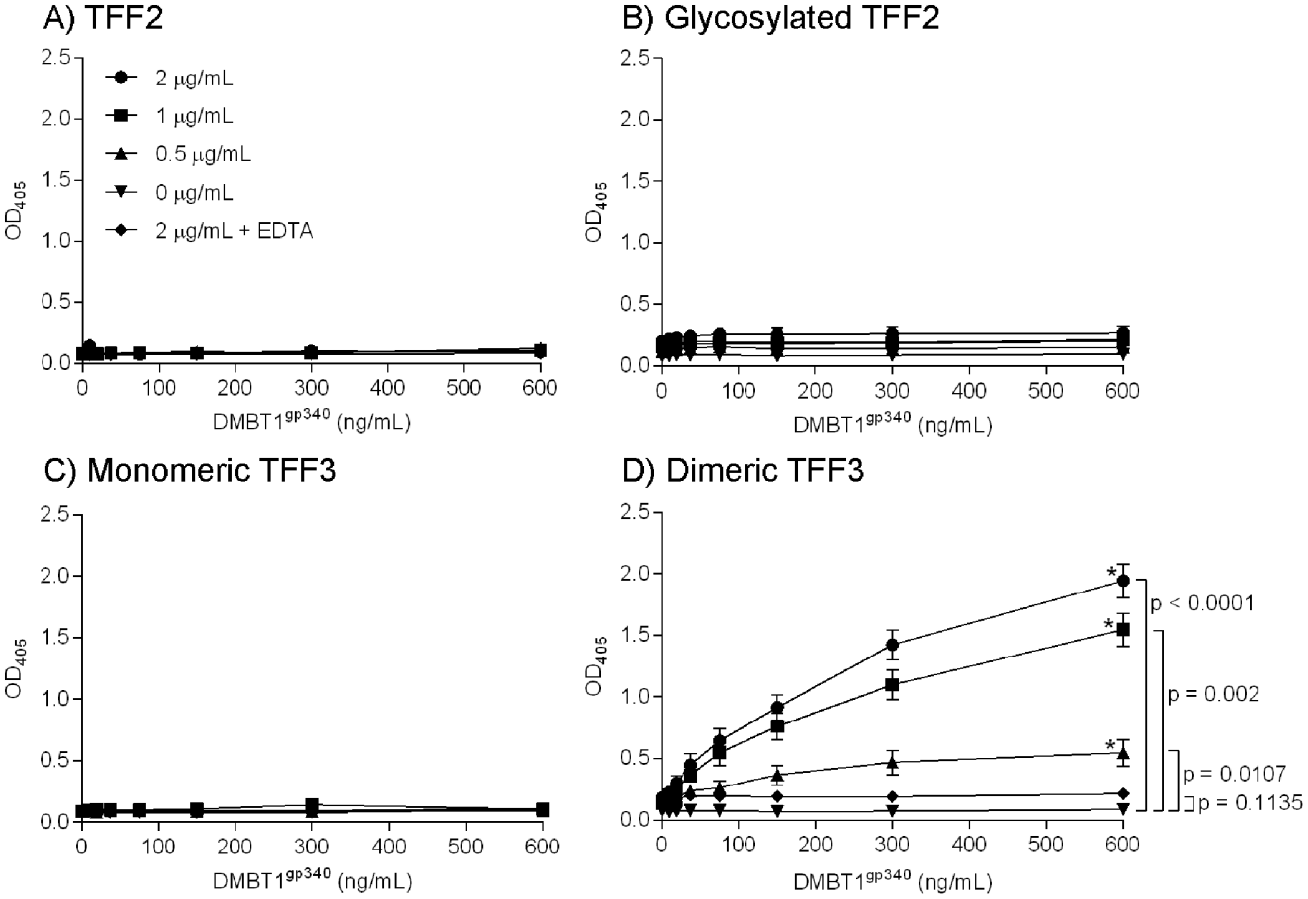
Binding of immobilized TFF proteins to DMBT1^gp340^. Serial diluted deglycosylated TFF2, glycosylated TFF2, monomeric TFF3 and dimeric TFF3 was coated to ELISA plates. The interaction was tested using two-fold serial diluted concentration of DMBT^gp340^ starting from 600 ng/mL. The binding of DMBT1^gp340^ was detected using a polyclonal rabbit anti-gp340 antibody (K421) as described in the Materials and Methods section. Serial dilution of recombinant TFF protein: 2 µg/mL (⧫), 1 µg/mL (▪), 0.5 µg/mL (▴), 0 µg/mL (•) and 2 µg/mL in EDTA (x). Each sample was done in duplicates and performed independently three times. Representative results are shown.

Binding of trefoil factors to DMBT1^gp340^ was analyzed using a solid-phase assay where plates were coated with recombinant forms of trefoil peptides: Glycosylated or non-glycosylated TFF2, monomeric TFF3 or dimeric TFF3. Binding of DMBT1^gp340^ to these peptides was detected using the polyclonal antibody K421 raised against DMBT1^gp340^ as detector and a secondary antibody conjugated with alkaline phosphatase and substrate for color indication. DMBT1^gp340^ did not bind either form of TFF2 proteins or monomeric TFF3 (**Fig. 6A–C**). However, DMBT1^gp340^ interacted with dimeric TFF3 in a concentration dependent manner and the binding was reaching near saturation when using 600 ng/mL DMBT1^gp340^, which was the highest concentration of DMBT1^gp340^ tested (**Fig. 6D**). No binding was seen in the presence of EDTA (**Fig. 6D**).

Statistical analysis showed that interaction between DMBT1^gp340^ and the dimeric TFF3 protein reached statistical significance in all TFF3 concentrations tested when compared to the control group with no dimeric TFF3 protein present (**Fig. 6D**). When testing the highest concentration of dimeric TFF3 protein in the presence of EDTA low binding values close to the binding values with no TFF3 protein present were seen (**Fig. 6D**). This was also reflected in the statistical analysis where no statistical significance was found between the two groups (p = 0.1135, **Fig. 6D**).

## Discussion

We have previously used Hyb213-1 for immunohistochemical localization of DMBT1^gp340^ and shown that it reacts with alveolar macrophages, other tissue macrophages, and with ducts of parotid glands but no reaction was seen in the small intestine [6]. This was in strong contrast to the finding of a very strong signal for DMBT1^gp340^ when analyzing for expression using RT-PCR [6]. Furthermore, a polyclonal anti-gp-340 antibody (K421) reacted strongly with epithelial cells in the small intestine [6]. These results prompted us to make new monoclonal antibodies directed against DMBT1^gp340^ and one of the resulting antibodies was Hyb213-6.

When evaluating the two monoclonal antibodies in Western blot analysis of crude lung washing showed that in a non-reduced state both Hyb213-6 and Hyb213-1 reacted with a double band of 290 kDa and when detergent solubilized alveolar macrophages from the same person were analyzed in parallel, a similar pattern was observed. Amino acid sequence of peptides derived from these bands has shown that they both were encoded by the *DMBT1* gene [16]. When analyzing saliva by Western blot analysis in the non-reduced state both antibodies reacted against the same bands as found for lung lavage. This finding was supported by immunohistochemical analysis of human submandibular glands where both antibodies showed staining of the serous acini and demilune cells. This is in accordance with previous results [41]. When analyzing colon extract in a non-reduced states Hyb213-6 reacted with a double band of approximately 270 kDa, whereas these bands were not recognized by Hyb213-1. Hyb213-1 has been shown to react with an O-linked sialidase-sensitive carbohydrate structure on DMBT1^gp340^ and cross-reacting with MUC7 [41]. Expression of MUC7 is restricted to the oral cavity and the respiratory tract [42], [43]. This implies that DMBT1^gp340^ expressed in these tissues could have a tissue specific glycosylation explaining why Hyb213-1 only reacts with DMBT1^gp340^ from these tissues and not with DMBT1^gp340^ from the small or large intestines (**Fig. 3** **and** **4**). Hyb213-6 has been shown to react with an epitope on DMBT1^gp340^ that is lost during reduction of the disulphide bridges in the molecule [41]. We find the same result for Hyb213-6 in this paper (and unpublished results) and this indicates that the epitope for Hyb213-6 is a structural dependent protein epitope that is lost during the unfolding of the protein under reducing conditions but present in all DMBT1 proteins investigated from the gastrointestinal and respiratory tract (**Fig. 1** **–** **3**).

The two forms of DMBT1^gp340^ found in lung washings clearly differ from the two forms found in the colon extract by molecular mass. They likely differ in their glycosylation pattern as studies, using mass spectrometry, of purified DMBT1^gp340^ from lung, saliva or the proximal or distal colon, respectively, have shown differential tissue specific glycosylation [44], [45]. However, the difference in molecular mass seen here between lung lavage and colon extract DMBT1^gp340^ (**Fig. 1**) and previous studies could simply be due to an inter-individual difference, as purified DMBT1^gp340^ isolated from saliva from three different individuals showed variance in size and glycosylation pattern [45]. At present it is not known if this difference in size is due to tissue specific glycosylation or inter-individual glycosylations as no study has looked at DMBT1 proteins from different tissue or body fluids from the same person.

Native DMBT1^gp340^ purified from BAL migrated under reducing SDS-PAGE conditions as two bands (band A and B, **fig. 5A**). This is in agreement with what we have previously found [16]. Purified recombinant TFF proteins migrated under reducing conditions with molecular weights of approximately 12 kDa (non-glycosylated TFF2), 15 kDa (glycosylated TFF2) and 7 kDa (monomeric and dimeric TFF3). This is also in agreement with previous findings where the mass of the proteins were identified by mass spectrometry [31]. The dimeric form of TFF3 has found to be held together by a disulphide bridge between two monomeric TFF3 molecules [33]. The disulphide binding will be reduced under reducing conditions and result in two monomeric forms of TFF3.This complies with what we observe here, where a shift in the molecular weight of dimeric TFF3 from non-reduced SDS-PAGE to reduced SDS-PAGE was observed and both monomeric and dimeric TFF3 show the same molecular weight under reducing conditions (**Fig. 5**).

We find here that the DMBT1 interaction with dimeric TFF3 is calcium-dependent. This calcium dependency has also been reported for the DMBT1 interaction with other secreted proteins such as SP-A, SP-D, MBL and secretory IgA [16], [17], [46], [47]. It is somewhat surprising that the two porcine and human DMBT1 protein homologues display a different binding specificity with regards to TFF proteins. The porcine DMBT1 was isolated from stomach scrapings. We have previously shown that the stomach is the primary organ for the expression of human TFF2, where the expression level was found to be more than ten-fold higher than any other organ tested in an array of 20 different organs [34]. It is not known whether the DMBT1 protein from the stomach is different from DMBT1 proteins expressed in the lung or colon as no information has been published on this. However, human TFF3 has a wider expression profile on mucosal surfaces in general and similar to the expression profile found for human and mouse DMBT1 [6], [13], [34]. It remains an open question whether different forms of DMBT1 from different tissues interact with different TFF proteins? The pH values between the stomach and the rest of the gastrointestinal tract are different. The surface structure of dimeric TFF3 in solution, using nuclear magnetic resonance, followed by comparison to monomeric TFF3 and dimeric TFF1 and TFF2 using computer modeling showed that the proteins display surface differences outside the core regions [48]. These core regions are believed to provide the scaffolding of the TFF proteins, highlighting that the differences found between the proteins would dictate the functional specificities of each protein [48]. It is therefore tempting to speculate that the pH difference seen in the gastrointestinal channel could have an influence on the function of the different TFF proteins by changing the charge of amino and carboxylated surface residues. Here we have only tested the interaction between DMBT1^gp340^ and TFF proteins at a pH value of 7.4 but it would be interesting to see if differences would be observed at different pH values.

We showed that DMBT1^gp340^ was up regulated in inflammatory bowel disease using a monoclonal antibody raised against native DMBT1^gp340^ (**Fig. 3**). This is consistent with what is seen in the literature measuring both the mRNA level as well as the protein level seen in immunohistochemical analysis using other antibodies raised against DMBT1 [26], [27]. We found that DMBT1 and TFF proteins don’t co-localize in the same cells. Instead, DMBT1 was localized to epithelial cells and TFF proteins to goblet cells (**Fig. 3** **and** **4**). Despite this spatial separation, the proteins would come in close proximity to each other in the mucus, so that interactions could take place outside the cells. The spatial separation of protein synthesis might serve to prevent the formation of viscous mucus in secretory vesicles. DMBT1^gp340^ has recently been found to be associated with mucins in the respiratory tract [49]. Trefoil peptides are also found in the respiratory tract [34], [50], [51]. Trefoil peptides and DMBT1 may therefore associate with the gel-forming mucins and may form a gel-network providing an immobilized reservoir of protective factors. This finding may place DMBT in a central situation where it could orchestrate different responses dependent on the molecules it is interacting with as DMBT1 also has been found to bind to SP-A, SP-D, complement factor C1q, Mannose Binding Lectin (MBL), lactoferrin and secretory IgA – all molecules of innate immunity [16], [17], [46], [47], [52], [53]. It has been suggested DMBT1 also has a role in cell growth and differentiation [54] and in combination with the finding that DMBT1 also interacts with TFF proteins, which are also involved in cell growth and differentiation [55], highlights the potential multifunctional role of DMBT1 *in vivo*. Further research is required to elucidate the *in vivo* role(s) of DMBT1 and what function each form of this multifaceted molecule undertakes.

We conclude that differently sized forms of DMBT1 are expressed between tissues that are differentially recognized by monoclonal antibodies raised against DMBT1^gp340^. A form of DMBT1 expressed in colon tissue was found to be specific for the monoclonal antibody Hyb213-6 and was not recognized by the monoclonal antibody Hyb213-1. DMBT1 was strongly up regulated in inflammatory bowel disease such as UC and DC, in epithelial cells adjacent to the TFF3-producing goblet cells. Furthermore, DMBT1^gp340^ bound to recombinant dimeric TFF3 in a solid-phase ELISA assay, but not to monomeric TFF3 or glycosylated or non-glycosylated TFF2. As animal models have shown that lack of DMBT1 and TFF proteins increase the susceptibly to inflammatory bowel diseases, the DMBT1-TFF3 interaction may have a role in the homeostasis of the normal gastrointestinal tract and in inflammatory bowel diseases *in vivo*.

## Supporting Information

Figure S1 **Intensity of TFF3 staining in normal human colon and inflammatory bowel diseases.** **A**) A dilution series from 1∶10,000 to 1∶3,200,000 was performed on normal human colon tissue to identify a dilution that gave weak but specific staining of TFF3. Original magnification 100x. **B**) TFF3 staining (1∶400,000) in normal colon and the inflammatory bowel diseases ulcerative colitis and Crohn’s disease. Tissue sections were stained using the polyclonal antibody raised against TFF3 and an indirect immunperoxidase technique and counterstained with Mayer’s hematoxylin as described in the Materials and Methods section. A positive signal is shown by brown color. Original magnification: 100x and 400x. Black squares in 100x pictures show approximately location of 400x pictures.
(TIF)
