# Supplementary figures and images for: A Variant Form of the Human Deleted in Malignant Brain Tumor 1 (DMBT1) Gene Shows Increased Expression in Inflammatory Bowel Diseases and Interacts with Dimeric Trefoil Factor 3 (TFF3)
Source: PLoS One. 2013 May 15;8(5):e64441. doi: 10.1371/journal.pone.0064441 (PMC3654909; doi:10.1371/journal.pone.0064441)

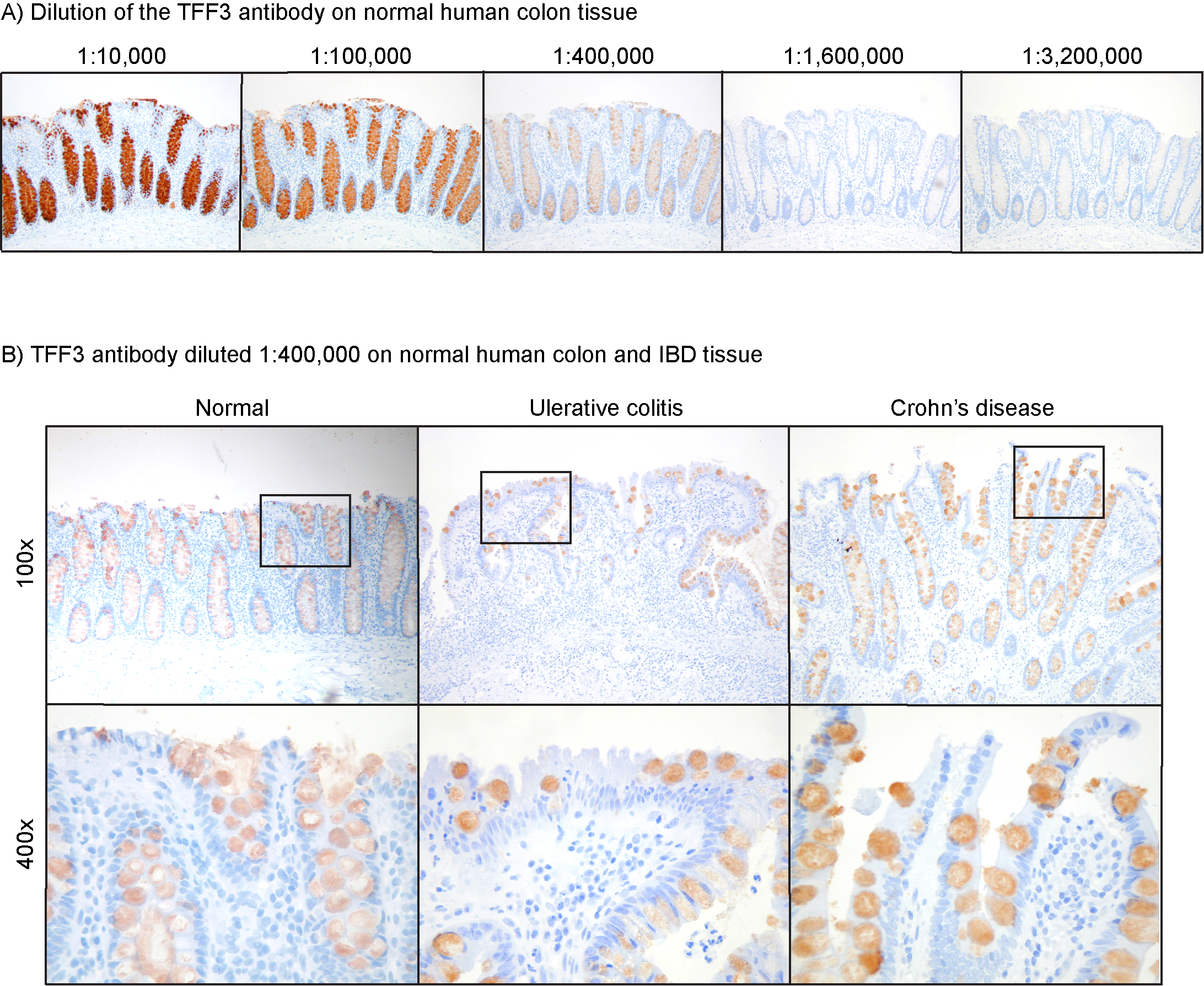

Supplement: Figure S1 — Intensity of TFF3 staining in normal human colon and inflammatory bowel diseases. A) A dilution series from 1∶10,000 to 1∶3,200,000 was performed on normal human colon tissue to identify a dilution that gave weak but specific staining of TFF3. Original magnification 100x. B) TFF3 staining (1∶400,000) in normal colon and the inflammatory bowel diseases ulcerative colitis and Crohn’s disease. Tissue sections were stained using the polyclonal antibody raised against TFF3 and an indirect immunperoxidase technique and counterstained with Mayer’s hematoxylin as described in the Materials and Methods section. A positive signal is shown by brown color. Original magnification: 100x and 400x. Black squares in 100x pictures show approximately location of 400x pictures. (TIF) [file pone.0064441.s001.tif]
